# Differential Effects of Monetary and Social Rewards on Product Online Rating Decisions in E-Commerce in China

**DOI:** 10.3389/fpsyg.2020.01440

**Published:** 2020-07-03

**Authors:** Cuicui Wang, Weizhong Fu, Jia Jin, Qian Shang, Xuan Luo, Xin Zhang

**Affiliations:** ^1^School of Management, Hefei University of Technology, Hefei, China; ^2^Key Laboratory of Process Optimization and Intelligent Decision-Making, Ministry of Education, Hefei University of Technology, Hefei, China; ^3^School of Business and Management, Shanghai International Studies University, Shanghai, China; ^4^Academy of Neuroeconomics and Neuromanagement, Ningbo University, Ningbo, China; ^5^Center of Group Behavior and Social Psychological Service, Ningbo University, Ningbo, China; ^6^School of Management, Hangzhou Dianzi University, Hangzhou, China

**Keywords:** monetary reward, social reward, event-related potentials, product rating decision, compensation effect

## Abstract

Humans can change their behaviors to obtain environmental rewards (e.g., money, food, and sex). However, our knowledge regarding how rewards affect human behaviors by priming and whether there are differences among types of rewards is limited. This study focused on whether monetary and social rewards have different priming effects on product rating decisions in e-commerce by using a behavioral experiment and event-related potentials (ERPs). Using cash/discount coupons as a monetary reward and greeting cards as a social reward, the behavioral data showed that unsatisfactory products with a monetary reward induced a less negative consumer attitude than those with a social reward or no reward; additionally, such products were associated with a longer reaction time while rating products than those with a social reward, reflecting that monetary rewards made it more difficult for the subjects to rate unsatisfactory products than social rewards. The P2, N2, and P3 components of the ERP data were evaluated. Unsatisfactory products caused negative emotion, which could be compensated more by the monetary reward than the social reward as reflected by a smaller P2 amplitude. Due to the compensation effect of the monetary reward, unsatisfactory products were associated with more decision conflict than the social reward as reflected by a more negative N2 amplitude, which is consistent with the behavioral results. However, in the subsequent controlled process, regardless of whether the products were satisfactory or unsatisfactory, the monetary reward caused more attention reallocation and was more motivating than the social reward as reflected by a larger P3 component. These findings have implications for the marketing strategy of online sellers and value of online reviews and suggest attaching importance to ethical issues induced by monetary rewards in rating behaviors.

## Introduction

Human behaviors can be affected by various types of environmental rewards, such as food, sex, money, or social affiliation (Ait Oumeziane et al., 2017; Wang et al., 2017). These rewards can meet individual needs and provide subjective pleasure. In e-commerce, online sellers often provide various types of rewards (i.e., cash/discount coupons, giveaways, and free sample). Thus, it is important for sellers to know the effects of different types of rewards on consumers’ attitudes and behaviors. The role of monetary rewards as a powerful incentive measure is well known. Recently, researchers have started to explore the differences in neural mechanisms between monetary and social rewards, and most research has focused on the different incentive effects of monetary and social rewards occurring after subjects achieve a certain goal-directed behavior (Izuma et al., 2008; Kohls et al., 2009; Spreckelmeyer et al., 2009; Flores et al., 2015; Ait Oumeziane et al., 2017). However, in real life, we often provide rewards without any direct requirements. For example, customers may receive smiles or coupons from staff members at a supermarket, and such rewards are given directly with no other requirement that may affect individual behaviors. However, knowledge regarding the priming effects of monetary and social rewards is limited.

In e-commerce in China, online sellers often send coupons (monetary reward) or greeting cards (social reward) to customers with no direct requirements to influence customer satisfaction and behavioral intention. Consumers’ behavioral intentions usually contain the following two aspects: willingness to recommend behavior and future purchasing behavior (Maulisa and Hijrah Hati, 2019). On the one hand, online sellers mail coupons or greeting cards with products purchased at online stores through an express company. Online sellers hope that the monetary or social rewards will increase customer satisfaction and lead to positive reviews of the purchase as customer-generated review ratings have a substantial impact on the success or failure of a product on internet commerce (Chevalier and Mayzlin, 2006; Lafky, 2014). On the other hand, in daily life, coupons and greeting cards are still repeatedly sent to customers by text or e-mail, which could serve as reminders to customers and affect purchase decisions in the future. Thus, monetary and social rewards are often used as primers in e-commerce platforms. However, few studies investigated whether monetary and social rewards have different priming effects on the decisions of customers to provide online review ratings in e-commerce, especially when customers have different degrees of customer satisfaction with the products purchased in online platforms.

## Conceptual Framework

### Product Rating Generation Process

A product’s star rating is a main online review element affecting product sales and consumers’ purchase intention (Luca, 2011; Moe and Trusov, 2011). Thus, it is necessary to know the online product rating generation process. First, previous research found that consumer preferences affected their rating choice and social interactions had an effect on the review generation process (Lee et al., 2015), providing empirical evidence enhancing our understanding of how social imitation and learning affect consumer rating generation. Second, recent studies found evidence of fake reviews in many contexts (Lappas et al., 2016; Wang et al., 2018). For example, in the context of hotel reviews, manipulating online reviews had a significant effect on changing product visibility (Lappas et al., 2016). In the online shopping context in China, the strategy of returning cash coupons if consumers give a five-star rating is likely to increase false rating behaviors (Wang et al., 2018). Third, the previous literature studied reviewer motivation and found that intrinsic and extrinsic factors, such as the social network structure, social interaction, economic incentives, etc., motivated reviewers to comment (Goes et al., 2014; Warut et al., 2018). Warut et al. (2018) found that money, which is an extrinsic reward, could attract new reviewers to give more positive reviews but reduced the participation level of existing reviewers.

Considering that social interactions with friends or a crowd can affect the review generation process (Lee et al., 2015), we speculated that social interaction between consumers and online sellers could affect the product rating generation process. Social rewards (e.g., smiles and greeting cards) are important mediums that could have an effect on the product rating generation process. In addition, monetary rewards during the feedback stage can lead to false-positive rating behaviors (Wang et al., 2018). We speculated that monetary rewards during the priming stage might affect the product rating generation. Thus, we examine prior works investigating the impact of monetary and social incentives in related contexts and speculate regarding the possible psychological mechanism driving consumers to give ratings for online products with monetary or social rewards.

### Impact of Monetary and Social Rewards

Monetary and social rewards are the two main reward types and are generally considered related to human motivation and behavior (Wang et al., 2017). Monetary rewards, such as discounts or coupons, tend to “serve as a means to an end” (Lea and Webley, 2006) and are valued for the economic advantages they offer to customers. Economic approaches to money are based on a model of rational behavior and are considered at the macroeconomic level of analysis. However, psychological approaches to money typically pay attention to human attitudes or related behaviors in special situations (Lea et al., 2009). Numerous studies indicate that money is not only instrumental but also symbolic and emotional in interpersonal and intrapersonal regulation (Zhang, 2009; Zhou et al., 2009). Social rewards, such as smiling faces, encouraging gestures, and verbal praise, are regarded as another essential and advanced reward process with a large impact on individuals’ behavioral development. Previous studies show that social rewards are positive reinforcers that can increase the likelihood that a corresponding behavior will be executed in the future (Spreckelmeyer et al., 2009; Stavropoulos and Carver, 2013). Moreover, neuroscience approaches are used to investigate the neural basis of monetary and social reward processing. Focusing on the motivation for goal-directed behavior, Izuma et al. (2008) used functional magnetic resonance imaging (fMRI) experiments to investigate whether the acquisition of a social reward activated the same reward-related brain areas as a monetary reward (Izuma et al., 2008). Spreckelmeyer et al. (2009) further explored the differences between men and women in coding monetary and social rewards at the brain level and found that monetary rewards evoked a wider network of mesolimbic brain regains than social rewards in men but not in women (Spreckelmeyer et al., 2009). Based on electrophysiological evidence, Flores et al. (2015) and Ait Oumeziane et al. (2017) used monetary and social incentive delay (MID and SID) tasks to study the different neural responses to anticipation and evaluation of monetary and social rewards (Flores et al., 2015; Ait Oumeziane et al., 2017).

In most previous research, monetary and social rewards were given as outcomes after individuals made certain behavioral decisions, and reward processing focused on the behavioral influencing mechanism from the perspective of incentive theory. However, whether differences exist between social and monetary rewards as priming stimuli in e-commerce, especially the effect of different types of rewards on individuals’ perception of dissatisfaction with products and subsequent review rating decision making, is unclear. The theory of planned behavior (TPB) developed by Ajzen aims to predict or understand actualized behaviors in specified circumstances and emphasizes the role of the following three concepts: attitudes toward a behavior, subjective norms, and perceived behavioral control (Ajzen, 1991). Based on this theory, a person who has a positive attitude receives great support from significant others and perceives that strong behavior control is more likely to perform a behavior. Bittner and Shipper (2014) used the improved model of the TPB to predict the purchase intention of gamified products. Amini et al. (2014) revealed that rewards serving as an intervention factor had important relationships with individuals’ attitudes, subjective norms, and perceived behavioral control and affected motivations for particular behaviors. In fact, rewards also play a prominent role in online behavior, and we can use the TPB to speculate the possible effect of rewards on consumers’ online behavior. In the current study, consumers have different emotions toward satisfactory and unsatisfactory products, which could affect their rating behavior. Unsatisfactory products could induce unpleasant feelings among consumers, which could be affected by different reward types. We speculate that monetary or social rewards given after customers experience dissatisfaction with products could compensate for the unpleasant feelings experienced by individuals, change their attitude, and further affect their review rating, which could represent a new perspective enhancing our understanding of reward processing.

The priming effect of money suggests that counting money beforehand can protect people from experiencing unpleasant feelings caused by physical pain or distress (Zhou et al., 2009). However, few studies have examined the priming effect of social rewards. In the present study, using the context of Business-to-Customer (B2C) e-retailing, we focus on the priming effect of monetary and social rewards on consumer-generated ratings of unsatisfactory and satisfactory products purchased by customers in e-commerce. In prior studies, monetary outcomes were reflected by wins or losses of money, and most social outcomes were reflected by individual face pictures/videos (i.e., a face with a slight smile) (Spreckelmeyer et al., 2009; Flores et al., 2015; Bottini, 2018). However, Yang et al. (2002) demonstrated that our brain is sensitive to facial stimuli, and thus, social reward processing may be conflated by face processing when using faces as social feedback. In research comparing the time courses of social and monetary reward processing, Ait Oumeziane et al. (2017) used a thumbs up or down to reflect social reward (Ait Oumeziane et al., 2017), which is perceptually not very similar to monetary reward because the material used as the social reward was a picture, while the monetary reward was in the form of several words. In e-commerce, cash/discount coupons and greeting cards are often shipped by merchants along with the products purchased from online stores^1^ (in China). Considering both the limitations of previous research concerning social rewards and the context of our study, we sought to use cash/discount coupons and greeting phrases as novel monetary and social rewards. To minimize the neural differences driven by the physical characteristics of both reward types (e.g., images of faces/thumbs vs. money), both reward types were displayed as Chinese characters and represented ecologically valid monetary and social stimuli, which could be effective for tapping into relevant real-world processes of e-commerce.

### ERP Method and ERP Components

An event-related potential (ERP) is an electrophysiological brain signal associated with cognitive responses to an event (e.g., the presentation of a stimulus). Recently, the ERP approach has begun to be used to measure the complex cognitive processes of consumer behavior in marketing (Telpaz et al., 2015; Venkatraman et al., 2015; Barnett and Cerf, 2017; Lin et al., 2018; Fu et al., 2019). To explore the potential neural processes of how different reward types have a priming effect on rating decision making for unsatisfactory products purchased by customers in e-retailing, we attempted to apply ERPs with a behavioral method to examine the dynamic electrophysiological time course. Flores et al. (2015) and Ait Oumeziane et al. (2017) used MID and SID tasks and proposed temporal stage models to describe monetary and social reward processing. During the reward anticipation stage, the N1, P2, and P3 components were found to reflect the allocation of attentional and motivational resources (Flores et al., 2015; Ait Oumeziane et al., 2017). During the reward evaluation stage, the P2, feedback-related negativity (FRN) and P3 components, which reflected affective and cognitive processes, were modulated by the reward types (Flores et al., 2015). In addition to the anticipation and evaluation stages, Ait Oumeziane et al. (2017) further studied the processing stage of reward cues. In the current study, monetary and social rewards were given to individuals as primers without any requests; thus, there was no reward cue stage or reward anticipation stage. We focused on the neural processes of different reward types affecting rating behavior when customers felt that the products or services purchased via e-commerce were unsatisfactory. Thus, the evaluation and decision processes were emphasized. Considering that the rewards in the current study were not provided during the feedback stage, we speculated that the FRN component would not be evoked. Based on previous ERP studies (Flores et al., 2015; Ait Oumeziane et al., 2017), three components, namely, P2, N2, and P3, could be involved in the temporal course of consumer-generated ratings of unsatisfactory products with two types of reward primers.

#### P2

The P2 component is a positive potential over the frontal region that occurs approximately 200 ms after the stimulus onset. Prior studies have suggested that the P2 component is related to the emotional evaluation of prospective rewards (Doñamayor et al., 2012; Flores et al., 2015) and that the P2 amplitude is associated with reward sensitivity (Martin and Potts, 2004; Potts et al., 2006). In the study conducted by Flores et al. (2015), there was no significant main effect of reward type on P2, but the P2 difference between reward and non-reward under the monetary condition was larger than that under the social feedback condition (Flores et al., 2015). In the current study, unsatisfactory products or services induced unpleasant feelings that could be compensated by monetary or social rewards, which could affect the emotional evaluation processes of rewards. Therefore, we speculate that emotional evaluation processes of different reward types could be affected by unpleasant feelings evoked by unsatisfactory products, which could be reflected by the amplitude of the P2 component.

#### N2

The N2 component is a negative component with a wave peaking at approximately 200–350 ms after stimulus onset, and N2 over the frontal region elicited by visual stimuli can be related to the detection of mismatch and conflict-related monitoring (Donkers and Van Boxtel, 2004; Folstein and Van Petten, 2008; Larson et al., 2014; Deng et al., 2015). Research has demonstrated that the amplitude of N2 in conflict detection is more negative (i.e., larger) in incongruent trials than in congruent trials (Veen and Carter, 2002; Yeung et al., 2004). Wang et al. (2018) found that the N2 component could reflect the perceptual conflict detected by subjects asked to give a good comment for a defective product (Wang et al., 2018). Moreover, rewards can modulate the perceptiveness of and adaptations to conflict (Braem et al., 2012). In the current study, based on the TPB, individuals had a negative attitude while rating an unsatisfying product but a positive attitude while rating a satisfactory product. Thus, response conflict occurs when subjects decide to give a five-star rating to an unsatisfying product, which evokes the N2 component. The different reward types could have a priming effect on the response tendency and moderate the amplitude of N2. Therefore, we speculate that conflict is detected when giving a good rating to an unsatisfactory product and that the reward type could moderate the conflict perception as reflected by the N2 component.

#### P3

The P3 component is maximal over parietal sites and is a positive ERP component with a wave peaking at approximately 300–500 ms following stimulus onset (Polich and Kok, 1995). Many previous studies suggested that the P3 amplitude is sensitive to reward valence (e.g., reward/non-reward) and magnitude (e.g., large reward/small reward), which is associated with attentional resource reallocation when evaluating the motivational significance of stimuli (Nieuwenhuis et al., 2005; Schevernels et al., 2014; Wu et al., 2016; Zhang et al., 2017). In addition, considering the differences between monetary and social rewards, Flores et al. (2015) found that monetary rewards might be more motivationally salient than social reward, which could be reflected by the P3 amplitude (Flores et al., 2015). In the current study, according to the TPB, there was a positive attitude toward satisfactory products and a negative attitude toward unsatisfactory products, which affected the motivational effects of the different reward types. Therefore, we speculated that the incentive effect of monetary and social rewards could be affected by the different attitudes caused by satisfactory or unsatisfactory products or services, which could be reflected by the amplitude of the P3 component.

The objective of this paper was to explore the neural processing of monetary and social rewards as primers in the decision to give review ratings for satisfactory or unsatisfactory products in e-commerce by using behavioral and ERP measures. We predicted that monetary and social rewards could have a significant effect on consumers’ attitude and rating behavior and that the amplitudes of the P2, N2, and P3 components could be evoked to reflect the neural processes. As mentioned above, P2 was expected to reflect the early emotional evaluation processes of different reward types, N2 was expected to reflect response conflict detection when giving review ratings for satisfactory or unsatisfactory products with different rewards as primers, and P3 was expected to reflect the incentive effect of monetary and social rewards in giving review rating decisions. The findings of this study could extend the current understanding of the different effects of reward types on consumer rating behavior and introduce the ERP method to thoroughly explore the neural bases above. In addition, this research aims to provide recommendations for e-retailers regarding how to use rewards as primers to exert positive influences on rating behavior.

## Study 1: ERP Experiment

### Materials and Methods

#### Participants

In the ERP experiment, ERP data from 21 native Chinese undergraduates (10 male) from Ningbo University aged between 20 and 26 years (*M* = 22.857, *S.D.* = 1.236) were analyzed in the current study. All participants were right-handed and had normal or corrected-to-normal vision with no history of neurological or psychiatric disorders. All participants had experience with online shopping and were familiar with cash/discount coupons and greeting cards in e-commerce. This study was approved by the Internal Review Board of the Center for Management Decision and Neuroscience at Ningbo University. Before the experiment, each participant provided written informed consent. The EEG data of the 1st, 11th, and 21st participants were excluded due to excessive artifacts. In total, 18 (9 male) subjects had valid behavioral data and EEG data.

#### Stimulus Materials

In the current ERP experiment, we used a priming-probe paradigm. In the priming set of stimuli (S1), each stimulus consisted of a product photo with a phrase below that described the perceived quality of the product by customers. Considering that experience products can be evaluated only during or after consumption while search products are evaluated prior to their purchase (Franke et al., 2004; Jiménez and Mendoza, 2013), experience products were more suitable as stimuli in the current experiment. Furthermore, the clothing category of experience products was chosen because youngsters attending a university generally have experience with buying clothing online and care about the impression made by clothing (Dhiman et al., 2018). Subsequently, to avoid differences in familiarity with the products between the men and women, a focus group discussion was conducted, and sweaters and shoes from the clothing category were chosen as the two products used in the current experiment. In addition, to control for physical features, such as color and style, we separately chose pictures of a sweater and a pair of shoes from the best-selling products of Taobao e-commerce^1^ as the stimuli for the two product pictures. According to the online comments of the two products, word frequency statistics were adopted to identify five phrases reflecting consumer satisfaction (e.g., works excellent) and five phrases reflecting consumer dissatisfaction (e.g., works rough). Therefore, S1 comprised 20 stimuli, i.e., 2 pictures (sweater or shoes) × 2 categories of product customer satisfaction × 5 phrases per category of product customer satisfaction.

The second set of stimuli (S2) comprised eight rewards (no more than seven Chinese characters) chosen from two categories, namely, monetary rewards and social rewards. The monetary rewards were reflected by four cash or discount coupons as follows: a 5 RMB cash coupon, a 10 RMB cash coupon, a 12% discount, and a 2% discount; these monetary rewards were frequently received by the customers in our pilot investigation. The social rewards were reflected by four greeting cards (e.g., enjoy your shopping). Therefore, the stimuli in the current experiment consisted of 160 pairs of product customer satisfaction (S1) and rewards (S2), i.e., 2 pictures (sweater or shoes) × 2 categories of product customer satisfaction × 5 phrases per category of product customer satisfaction × 2 types of rewards × 4 rewards per reward type. Each stimulus was digitized to 400 pixels × 300 pixels, and the mean luminance level of the stimuli was 186.17 cd/m^2^ (candela/square meter) with a standard deviation of 25.82 cd/m^2^.

#### Procedures

The ERP experiment comprised four blocks, and each block included 40 pairs of product pictures with product customer satisfaction (S1) and rewards (S2). All pair sequences were randomized. The presentation of all stimuli was controlled by the E-prime 2.0 software package (Psychology Software Tools, Pittsburgh, PA, United States). Each stimulus was presented at the center of a computer screen placed 1 m away from the eyes of the participants. Thus, the horizontal and vertical visual angles were 2.58° and 2.4°, respectively.

**FIGURE 1:**
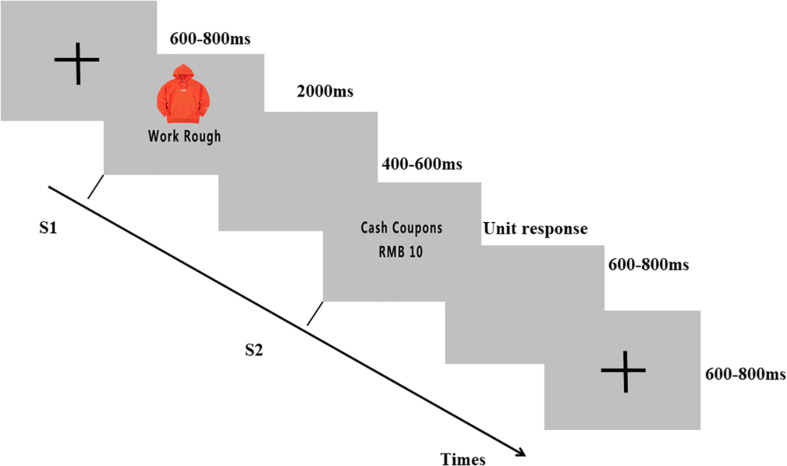
Event-related potentials (ERP) paradigm. Participants were instructed to observe a product photo with a phrase below that described the perceived quality of the product by customers (S1) and then decide whether to give the product a five-star rating with the presentation of monetary or social rewards (S2). Subject EEGs were recorded throughout the experiment.

In each trial, a fixation cross “+” was presented against a gray background for 600–800 ms. Then, S1 was presented for 2000 ms, followed by a blank screen for 600–800 ms; then, S2 appeared. After the participants made decisions, S2 disappeared, followed by a blank screen for 600–800 ms (as shown in Figure 1).

The participants were seated in a sound-attenuated and electrically shielded room and were required to fix their eyes on the fixation cross at the center of the screen. The participants were given the following introductions: “You received a parcel that included a sweater/a pair of shoes bought from Taobao.com, and the quality may or may not be to your satisfaction, which is reflected by S1. In addition, a coupon or a card was mailed with the product, which is reflected by S2. According to the information of S1 and S2, please evaluate whether you would give the product a five-star rating or not.” A compatible keyboard was used to allow the participants to make their behavioral decisions. Eleven subjects were asked to press 1 to indicate their choice to give a five-star rating and 3 to indicate their choice to not give a five-star rating. For the other 10 subjects, the keys were defined in reverse to counterbalance the difference between left and right hands. After the introduction, 10 training trials were performed by each participant as practice trials. The participants could rest for several minutes after each block. After the end of the experiment, the participants were paid 50 Chinese yuan (approximately US$ 7) for their participation.

#### Electroencephalography Recordings and Analysis

Electroencephalography (EEG) was recorded using a NeuroScan SynAmps2 Amplifier (Curry 7, Neurosoft Labs, Inc., Sterling, VA, United States), which harbored 64 Ag/AgCl electrodes according to the extended international 10–20 system and had a sampling rate of 1,000 Hz. The EEGs were referenced to the left mastoid with a cephalic (forehead) location as the ground. Electrooculogram (EOG) electrodes placed 10 mm from the lateral canthi of both eyes and above/below the left eye recorded blinks and vertical/horizontal eye movements, respectively. The experiment began when the electrode impedances were kept under 10 kΩ.

The EEG recordings were processed offline using NeuroScan analysis software (Scan 4.5, Neurosoft Labs, Inc., Sterling, VA, United States). The EOG artifacts (eye blinks and movement) were corrected. The EEG signals were digitally filtered through a zero-phase shift (low pass at 20 Hz, 24 dB/Octave) and divided into epochs extending from 200 ms before the onset of S2 to 800 ms after S2 onset with a 200 ms period prior to S2 onset as baseline correction. The trials during which the peak voltages exceeded±100 μV after correction were excluded before averaging. More than 30 sweeps in each condition remained, which was adequate for achieving stable and reliable measurements of P2, N2, and P3 (Luck, 2005). Thus, the EEG data of three participants (the 1st, 11th, and 21st participants) were excluded due to the attainment of less than 30 valid trials per condition. The EEG epochs of each subject were averaged across the four conditions (2 categories of product customer satisfaction × 2 categories of reward type).

Consistent with the published guidelines mentioned in the Section “Introduction,” three ERP components, namely, P2, N2, and P3, were analyzed in the current experiment. Based on a visual inspection of the grand-average data and previous research cited in the Section “Introduction,” the following representative channels and time windows of P2, N2, and P3 were selected: (I) P2, channels F3, Fz, F4, FC3, FCz, and FC4 in the time window from 200 ms to 260 ms; (II) N2, channels F3, Fz, F4, FC3, FCz, and FC4 in the time window from 270 to 370 ms; and (III) P3, channels P3, Pz, P4, PO3, POz, and PO4 in the time window from 300 to 450 ms. The mean amplitudes under each condition were extracted separately within the time windows of P2, N2, and P3. A three-way repeated-measures ANOVA with three within-subject factors (i.e., product customer satisfaction, reward type and electrode) was conducted for each component.

### Results

#### Behavioral Results

In the ERP experiment, to analyze the possible differences in favorite ratings (FRs) and reaction times (RTs) between monetary and social rewards following the presentation of satisfactory products or unsatisfactory products, two-way 2 (satisfactory products vs. unsatisfactory products) × 2 (monetary reward vs. social reward) repeated-measures ANOVAs were performed. Regarding the FRs, there were statistically significant differences between the satisfactory products and unsatisfactory products, *F*(1,17) = 1606, *p* < 0.001, η^2^ = 0.990, and the satisfactory products (*M* = 0.945, *S.E*. = 0.021) had higher FRs than the unsatisfactory products (*M* = 0.032, *S.E*. = 0.011). There was no significant difference between the monetary and social rewards, and no significant interaction effect was observed between product customer satisfaction and the reward type.

**FIGURE 2:**
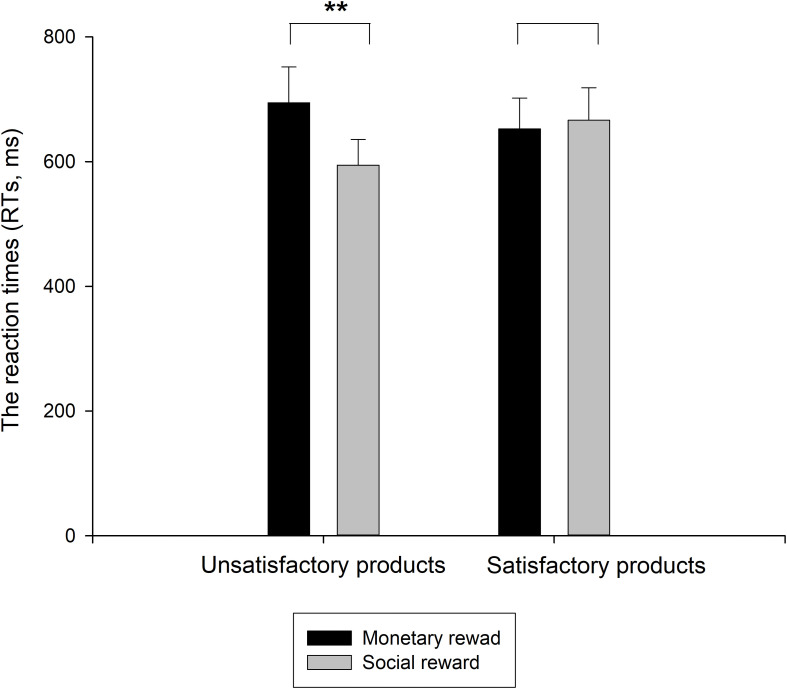
Mean RTs to the monetary and social rewards sorted by product customer satisfaction as satisfactory products and unsatisfactory products. Error bars indicate the SE of the RT. **p < 0.01.

Regarding the RTs in the ERP experiment, there was a significant effect of the reward type, *F*(1,17) = 4.932, *p* < 0.05, η^2^ = 0.225, and the monetary reward (*M* = 673.583, *S.E*. = 50.061) had a longer RT than the social reward (*M* = 630.426, *S.E*. = 44.589). There was no significant effect of product customer satisfaction (*p* > 0.1), but the interaction effect between the reward type and product customer satisfaction was notable [*F*(1,17) = 8.219, *p* < 0.05, η^2^ = 0.326]. Therefore, a simple effect analysis was conducted (as shown in Figure 2). Under the condition of satisfactory products, no significant effect was found between the monetary and social rewards (*p* > 0.1). However, under the condition of unsatisfactory products, the difference between the monetary and social rewards was significant [*F*(1,17) = 9.994, *p* < 0.01, η^2^ = 0.370], suggesting that consumers required a longer time to make a rating decision when presented with the monetary reward (*M* = 694.550, *S.E*. = 57.285) than when presented with the social reward (*M* = 594.524, *S.E*. = 41.158). In addition, a 2 (product customer satisfaction) × 2 (reward type) repeated ANOVA with gender as the between-subjects factor was conducted to analyze the RTs. There was no significant main effect of gender, and no significant interaction effects were observed between gender and product customer satisfaction/reward type or among all three factors above.

#### ERP Results

**FIGURE 3:**
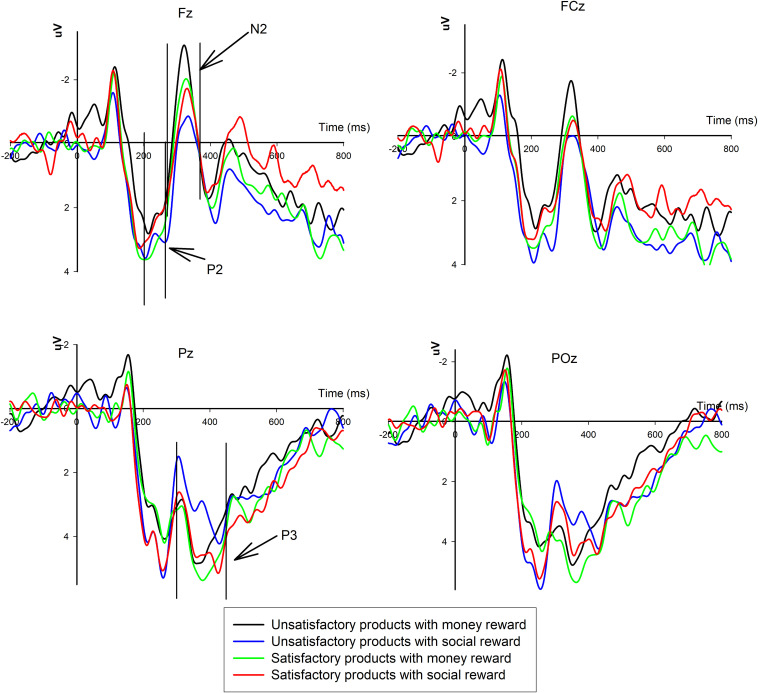
Event-related potentials grand-average waveforms elicited in response to different product customer satisfactions and reward types at channels Fz, FCz, Pz, and POz are presented.

The grand-average ERPs in response to the factors of reward type and product customer satisfaction are shown in Figure 3. Repeated-measures ANOVAs of P2, N2, and P3 were performed in three time windows.

A three-way 2 (product customer satisfaction: satisfactory vs. unsatisfactory products) × 2 (reward type: monetary vs. social rewards) × 6 (electrode: F3, Fz, F4, FC3, FCz, and FC4) ANOVA was performed to analyze the P2 component in the time window of 200 to 260 ms. There was no significant main effect of the reward type (*p* > 0.1), product quality (*p* > 0.1), or electrode (*p* > 0.1). However, the interaction effect between the reward type and product quality was notable, *F*(1,17) = 5.386, *p* < 0.05, η^2^ = 0.241. Thus, a simple effect analysis was conducted. Under the monetary reward condition, there were significant differences between the satisfactory and unsatisfactory products, *F*(1,17) = 4.641, *p* < 0.05, η^2^ = 0.214, reflecting that the satisfactory products (*M* = 3.182, *S.E.* = 0.782) evoked a larger P2 amplitude than the unsatisfactory products (*M* = 2.506, *S.E.* = 0.773) in processing monetary rewards. However, under the social reward condition, no significant difference was found between the satisfactory and unsatisfactory products (*p* > 0.1).

A 2 (product customer satisfaction: satisfactory vs. unsatisfactory products) × 2 (reward type: monetary vs. social rewards) × 6 (electrode: F3, Fz, F4, FC3, FCz, and FC4) ANOVA was conducted to analyze the N2 component in the time window of 270 to 370 ms. No significant main effect of product quality (*p* > 0.1) or reward type (*p* > 0.1) was found, but the interaction effect between product quality and reward type was significant [*F*(1,17) = 4.792, *p* < 0.05, η^2^ = 0.220]. Therefore, a simple effect analysis was conducted. Under the condition of satisfactory products, there was no significant difference between the monetary and social rewards (*p* > 0.1). However, under the condition of unsatisfactory products, the difference between the reward types was significant [*F*(1,17) = 5.298, *p* < 0.05, η^2^ = 0.238], suggesting that the monetary rewards (*M* = -0.480, *S.E*. = 0.821) elicited a more negative N2 amplitude than the social rewards (*M* = 0.538, *S.E*. = 0.846).

A 2 (product quality: satisfactory vs. unsatisfactory products) × 2 (reward type: monetary vs. social rewards) × 6 (electrode: P3, PZ, P4, PO3, POz, and PO4) ANOVA was conducted to analyze the P3 component in the time window of 300 to 450 ms. There was a significant effect of product quality [*F*(1,17) = 7.041, *p* < 0.05, η^2^ = 0.293], reward type [*F*(1,17) = 5.885, *p* < 0.05, η^2^ = 0.257], and electrode [*F*(5,17) = 9.890, *p* < 0.05, η^2^ = 0.128], but there was no significant interaction effect between product quality and reward type (*p* < 0.1). Regarding the product quality factor, the satisfactory products (*M* = 4.664, *S.E*. = 0.466) evoked a larger P3 amplitude than the unsatisfactory products (*M* = 4.049, *S.E*. = 0.361). Regarding the reward type, the monetary reward (*M* = 4.658, *S.E.* = 0.422) evoked a larger P3 amplitude than the social reward (*M* = 4.055, *S.E*. = 0.417).

In addition, 2 (product customer satisfaction) × 2 (reward type) × 6 (electrode) repeated-measures ANOVAs with gender as a between-subjects factor was conducted to analyze the P2, N2, and P3 components separately. The results showed that the main effect of gender on the P2, N2, and P3 amplitudes was not significant (*p* > 0.05), and none of the interaction effects between gender and product customer satisfaction/reward type or among all three factors above were significant (*p* > 0.05).

### Discussion

The ERP method was used in Study 1 to examine the potential neural processing of monetary rewards (cash/discount coupons) and social rewards (greeting cards) when customers were satisfied or dissatisfied with the products purchased in e-retailing. The behavioral results showed that the satisfactory products had a higher favorite rating than the unsatisfactory products and that the monetary reward was associated with a longer time required by the consumers to make rating decisions regarding the unsatisfactory products than the social reward. However, there was no significant difference between the monetary and social rewards, and no significant interaction effect was observed between product customer satisfaction and the reward type on the favorite rating behavior. A possible reason could be that the binary state of the choice (give or not give a five-star rating) in Study 1 had a low sensitivity and could not properly reflect the consumers’ real attitude. The participants may have positive/negative rating behaviors in response to both monetary and social rewards, but the degree of their attitude differed between the monetary and social rewards. In addition, the sample size could not adequately reflect the rating behaviors, and we would conduct Study 2 to further explore.

The ERP results demonstrated that monetary rewards for satisfactory products evoked a larger P2 amplitude than monetary rewards for unsatisfactory products, and no differences were observed in response to social rewards. However, monetary rewards for unsatisfactory products elicited a more negative N2 amplitude than social rewards. Regarding the P3 component, the amplitude evoked by satisfactory products and monetary rewards was larger than that evoked by unsatisfactory products and social rewards, and no interactive effects were observed between the products and reward factors.

Regarding the ERP results, we found that the P2 amplitude evoked by monetary rewards for satisfactory products was larger than that evoked by unsatisfactory products. As mentioned above, the P2 component is an early attention-related potential and is associated with the emotional evaluation of rewards (Ito and Urland, 2003, 2005; Potts et al., 2006; Doñamayor et al., 2012; Flores et al., 2015). In the current study, the unsatisfactory products induced negative feelings that could be offset by the subsequent monetary reward. Thus, under the condition of unsatisfactory products, the early emotional evaluation of the monetary reward was decreased, which was reflected by a less-positive P2 component. However, in response to the social reward, no significant difference in the P2 component was found between the satisfactory and unsatisfactory products. A possible reason is that the early emotional evaluation of the social reward was not affected by the negative emotion induced by the unsatisfactory products. Thus, the social reward was not considered compensation for the unsatisfactory products.

As partially expected based on the result of the N2 component, the monetary reward elicited a more negative N2 potential than the social reward under the unsatisfactory products conditions without salient differences in response to the satisfactory products. As mentioned in the Section “Introduction,” the anterior N2 component could reflect cognitive conflict monitoring in decision-making processes (Folstein and Van Petten, 2008; Larson et al., 2014; Deng et al., 2015; Shang et al., 2017). In addition, incorrect decision tendencies are overturned by overt correct decisions, resulting in high decision conflict, which could evoke the N2 component (Ridderinkhof et al., 2004). In the current study, the overt correct response to the unsatisfactory products was not giving a five-star rating, whereas giving a five-star rating could be considered an incorrect response. The monetary reward could counteract the negative effects of the unpleasant feelings caused by the unsatisfactory products, while the social reward could not be considered compensation for the unsatisfactory products. Thus, more decision conflict was detected when the subjects made the choice to give a five-star rating to the unsatisfactory products when offered a monetary reward than when offered a social reward; thus, the N2 component evoked by the monetary reward was more negative than that evoked by the social reward under the unsatisfactory products condition. However, no significant difference in the N2 component was found between the monetary reward and social reward under the satisfactory products condition. Notably, the stimuli used to present the monetary reward were discount coupons or cash coupons that could be spent only at specified online retailers rather than currency. This factor could have contributed to the lack of significant difference in conflict detection between the monetary reward and social reward under the satisfactory products condition.

The P3 component was found following the N2 component in this study. Regarding the P3 component, we found that the monetary reward evoked a larger P3 amplitude than the social reward and that the P3 elicited by the satisfactory products was more positive than that elicited by the unsatisfactory products. Previous studies have demonstrated that P3 can index the reallocation of attention and that a more positive P3 amplitude suggests that more attentional resources are paid to the stimuli and enhance the activation of the motivational system (Polich and Kok, 1995; Yeung and Sanfey, 2004; Broyd et al., 2012; Doñamayor et al., 2012; Wang et al., 2018). In the current study, the monetary reward reallocated more attention during the later processing stage and had a greater incentive effect on the subjects to make decisions than the social reward. In addition, product customer satisfaction could affect the later controlled and elaborate processing of the reward stimuli. According to the TPB, when the subjects received unsatisfactory products, a negative attitude toward giving five-star ratings was induced, and the motivation to give such products a favorable rating was lower than that when receiving satisfactory products. Thus, the P3 component evoked by the monetary reward and satisfactory products was larger than that evoked by the social reward and unsatisfactory products separately in the current study. However, there was no interaction effect between the factors reward type and product customer satisfaction in the P3 component.

## Study 2: Behavioral Experiment

In Study 2, we used a behavioral experiment and enlarge the sample size to probe the robustness of the effect observed in Study 1. First, to address the potential issue of the lack of a control, a control group receiving no reward type was tested in Study 2. Second, according to the theory of TPB, consumers’ attitudes were closely associated with their rating behavior. Thus, we used a seven-item scale to measure the consumers’ attitudes and their likelihood of giving five-star ratings across the conditions in Study 2 to further explain the behavioral results in the ERP experiment. Third, gender and online shopping experience were considered in Study 2. Therefore, Study 2 included some control variables and used a different method with a larger sample size to further explore the differential effects of monetary and social rewards on product online rating decisions in e-commerce.

### Materials and Methods

#### Participants

In the behavioral experiment, 365 native Chinese (84.7% 19–24 years old, 61.9% female) mainly from Hefei University of Technology and Ningbo University participated in this behavioral investigation research in exchange for monetary compensation, and none of these participants participated in the ERP experiment in Study 1. All participants had online shopping experience; 87.4% of the participants had more than 2 years of online shopping experience, and 95.1% of the participants engaged in online shopping at least once every 3 months. In total, 158 participants were subjected to the no-reward condition, 102 participants were subjected to the monetary reward condition, and 105 participants were subjected to the social reward condition.

#### Stimulus Materials

In the behavioral experiment, the materials selected were the same as those described in the ERP experiment. The behavioral experiment employed a 2 (product customer satisfaction: satisfactory or unsatisfactory) × 3 (reward type: monetary reward, social reward or no reward) mixed design. Product customer satisfaction was a within-subject factor, and the reward type was a between-subject factor. The monetary reward type, social reward type, and no-reward type conditions included sweaters and shoes from the clothing category with phrases reflecting consumer satisfaction or dissatisfaction. The monetary rewards were reflected by cash or discount coupons, while the social rewards were reflected by greeting cards, which were the same as those described in the ERP experiment.

#### Procedures

In the behavioral experiment, the participants in each reward type condition (monetary reward, social reward, and no reward) were informed that the research purpose was to understand how consumers give ratings to the goods purchased in online shopping. After the introduction, the participants imagined that they received a sweater/a pair of shoes purchased from Taobao.com, and the quality was reflected by the following pictures with phrases, which were the same as the pictures used in the ERP experiment. Moreover, under the monetary and social reward conditions, the information “online sellers gave cash or discount coupons/greeting cards as show in the following pictures” was presented. After reviewing the product information, the participants reported their attitudes toward this online shopping experience or online seller (1 = not at all pleasant, 7 = very pleasant; 1 = not at all satisfactory, 7 = very satisfactory; 1 = not at all reliable, 7 = very reliable; α = 0.95) and how likely they would be to give five-star ratings to this purchase (1 = not at all likely, 7 = very likely). Considering the high internal consistency (α = 0.95), we used the average to form a consumer attitude index.

### Results and Discussion

#### Results

For the behavioral experiment, two-way 2 (satisfactory products vs. unsatisfactory products) × 3 (monetary reward vs. social reward vs. no reward) repeated-measures ANOVAs were performed to analyze the attitudes toward this online shopping experience and rating behaviors. Regarding consumer attitudes, there were significant differences between the satisfactory products and unsatisfactory products, *F*(1,362) = 737.73, *p* < 0.001, η^2^ = 0.671, and consumers had more positive attitudes toward the satisfactory products (*M* = 5.443, *S.E*. = 0.064) than the unsatisfactory products (*M* = 2.980, *S.E.* = 0.071). The main effect of reward type was significant [*F*(2,362) = 16.223, *p* < 0.01, η^2^ = 0.082], and the consumer attitudes under the no-reward condition were lower than those under the monetary reward (*p* < 0.01) and social reward (*p* < 0.01) conditions. Moreover, the interaction effect between the reward type and product customer satisfaction was notable [*F*(2,362) = 10.256, *p* < 0.01, η^2^ = 0.054]. Therefore, a simple effect analysis was conducted, and under the satisfactory products condition, no significant effect was found among the monetary, social, and no-reward types (*p* > 0.05). However, under the unsatisfactory products condition, the difference among the three reward types was significant [*F*(2,362) = 22.246, *p* < 0.01, η^2^ = 0.109]. The consumer attitudes under the monetary reward type condition (*M* = 3.549, *S.E.* = 0.133) were less negative than those under the social reward (*M* = 2.971, *S.E.* = 0.131, *p* < 0.01), and no-reward (*M* = 2.421, *S.E.* = 0.107, *p* < 0.01) conditions, and the consumer attitudes under the social reward type condition were less negative than those under the no-reward type condition (*p* < 0.01). In addition, there were no significant main effects of gender or online shopping experience, and the interaction effects between gender/online shopping experience and product customer satisfaction or among the reward types on consumer attitude were statistically insignificant (*p* > 0.1).

Regarding the rating behaviors in the behavioral experiment, the satisfactory products (*M* = 5.439, *S.E.* = 0.074) were more likely to receive five-star ratings than the unsatisfactory products (*M* = 2.818, *S.E.* = 0.073, *p* < 0.01). The factor of the reward type had a significant main effect [*F*(2,362) = 5.419, *p* < 0.01, η^2^ = 0.029], and consumers were less likely to give five-star ratings under the no-reward condition (*M* = 3.894, *S.E.* = 0.084) than the monetary reward (*M* = 4.314, *S.E.* = 0.104, *p* < 0.01) and social reward (*M* = 4.179, *S.E.* = 0.103, *p* < 0.1) conditions. More importantly, the interaction effect between the reward type and product customer satisfaction was marginally significant [*F*(2,362) = 2.950, *p* < 0.1, η^2^ = 0.016]. Therefore, a simple effect analysis was conducted. Under the satisfactory products condition, no significant effect was found among the three reward types (*p* > 0.05). However, under the unsatisfactory products condition, the difference among the three reward types was significant [*F*(2,362) = 8.310, *p* < 0.01, η^2^ = 0.044], and the consumers were less likely to give five-star ratings under the no-reward condition (*M* = 2.456, *S.E.* = 0.108) than under the monetary reward (*M* = 3.147, *S.E.* = 0.135, *p* < 0.01) and social reward (*M* = 2.852, *S.E.* = 0.133, *p* < 0.1) conditions. However, the rating behaviors did not significantly differ between the monetary reward and social reward types (*p* > 0.1). In addition, there were no significant effects of gender or online shopping experience, and no significant interaction effects were observed between gender/online shopping experience and product customer satisfaction or among the reward types on the rating behaviors (*p* > 0.1).

#### Discussion

The behavioral experiment in Study 2 was conducted to examine the emotional effect of monetary rewards (cash/discount coupons) and social rewards (greeting cards) on consumers’ attitudes and rating behavior when the customers were satisfied or dissatisfied with the products purchased in e-retailing. According to the results of the main effect, the consumers had a more positive attitude toward and were more willing to give a five-star rating for satisfactory products than unsatisfactory products. Moreover, compared with the control group (no-reward condition), the consumers under the monetary and social reward conditions had a more positive attitude and preferred to give five-star ratings, reflecting that either monetary or social rewards could improve consumer attitudes and promote positive rating behavior.

Regarding the results of the interaction effect, the three reward conditions had different significant effects on consumer attitudes and rating behaviors only when the consumers received an unsatisfactory product. Thus, under the satisfactory products condition, the effect of the three reward conditions on consumer attitudes and rating behaviors did not significantly differ. Specifically, the monetary reward induced a more positive attitude than the social reward under the unsatisfactory products condition, indicating that monetary rewards can decrease the more negative feeling evoked by unsatisfactory products than social rewards. Moreover, the consumers under the no-reward condition were less likely to give five-star ratings than those under the monetary and social rewards conditions under the unsatisfactory products condition. The rating behaviors did not significantly differ between the monetary and social rewards under the satisfactory products condition (*p* = 0.120), but the average likelihood of giving a five-star rating under the monetary reward condition was larger than that under the social monetary reward condition. This finding may indicate that different effects of monetary and social rewards on rating behavior exist to some extent, and combining the reaction time results presented in Study 1 is needed to further explain this finding.

## General Discussion

### Research Finding

In the current study, we used behavioral and ERP experiments to examine the emotional effect and potential neural processing of monetary rewards (cash/discount coupons) and social rewards (greeting cards) when customers are satisfied or dissatisfied with products purchased in e-retailing.

The behavioral results of both Study 1 and Study 2 reflected that customers were more willing to give a five-star rating for satisfactory products than unsatisfactory products. According to the results of Study 1, customers had a more positive attitude toward satisfactory products than unsatisfactory products, and thus, the former had a higher rating than the latter, supporting the TPB. Regarding unsatisfactory products, monetary rewards induced more positive attitudes than social rewards and no rewards, while social rewards induced more positive attitudes than no rewards, indicating that monetary and social rewards can decrease negative feelings evoked by unsatisfactory products and that the compensation effect of monetary rewards could be better than that of social rewards. Moreover, according to the RT results in Study 1, the customers under the monetary reward condition took a longer time to make a decision than those under the social reward condition. Previous research has demonstrated that a longer reaction time is associated with a higher cognitive load and greater task difficulty (Sweller, 1988; Wang et al., 2016; Jin et al., 2017). In this study, unsatisfactory products and monetary rewards guided the decision to give a five-star rating in two opposite directions. The unsatisfactory products were related to negative attitudes and negative review ratings, while monetary rewards were related to positive attitudes and positive review ratings, thus increasing the decision difficulty. Thus, the participants needed to exert more cognitive effort to make the decision to give the product five stars when a monetary reward was offered for unsatisfactory products, whereas it was relatively easier to make a decision when a social reward was offered for unsatisfactory products. Thus, monetary rewards affect the decision process more than social rewards when customers are dissatisfied with the quality of a product.

In addition, the behavioral results of the ERP experiment in Study 1 were not exactly consistent with the results of the behavioral experiment in Study 2, which was reflected by the lack of an interaction effect on the rating behavior between the reward type and product quality. There are two possible reasons for the notable interaction effects. First, a no-reward condition as a control group was included in Study 2, which could have led to the differences in the interaction effects between the two experiments. Second, the binary state of the choice (giving or not giving a five-star rating) in Study 1 had a low sensitivity and could not reflect the consumers’ real rating attitude; in contrast, a seven-item scale was used to measure the likelihood of giving five-star ratings in Study 2. Moreover, the reaction time data recorded in Study 1 could further support the conclusions of Study 2.

Three ERP components were found to reflect the neural processing of the monetary and social rewards as primers in the decision to give review ratings for satisfactory or unsatisfactory products in e-commerce. Monetary rewards for satisfactory products elicited a more positive P2 amplitude than monetary rewards for unsatisfactory products, but no differences were observed in response to social rewards. However, monetary rewards for unsatisfactory products evoked a more negative N2 amplitude than social rewards. Regarding the P3 component, the amplitude evoked by the satisfactory products and monetary reward was larger than that evoked by the unsatisfactory products and social reward, and no interactive effects between the products and reward factors were observed. The significant interaction effects on the P2 and N2 components may suggest that unsatisfactory products affect the early reward processing stage, without any notable effect on the later elaborate process. Thus, the unsatisfactory products caused a negative emotion that could be compensated by a monetary reward (but not a social reward) as reflected by the P2 component. Thus, the unsatisfactory products with monetary rewards were observed to have greater decision conflict regarding whether to give a five-star rating than the products with social rewards as reflected by the N2 component. In the later controlled process, regardless of whether the products were satisfactory or unsatisfactory, the monetary rewards caused more attention reallocation and were more motivating as reflected by the P3 component. In addition, different rewards as primers influenced P2, N2, and P3, which may provide deep insight into these three components. It can be speculated that the P2, N2, and P3 components were not only sensitive to the rewards during the feedback stage but also evoked by the rewards during the priming stage.

Furthermore, the results show that there was no significant gender effect in the two experiments. In a study conducted by Spreckelmeyer et al. (2009) involving social and monetary rewards as feedback, a gender effect was found in the behavioral and brain data, demonstrating that males, but not females, are more sensitive to monetary rewards than social rewards. A possible explanation for the lack of a significant gender effect is that the paradigm in the current study used monetary and social rewards as primers, and the task was rating behavior in e-commerce. Therefore, males and females may have similar psychological and cognitive processing when facing different reward types in giving rating decisions. In addition, the current study did not find a significant difference in the online shopping experience factor in the behavioral experiment. A possible reason is that most participants had relatively rich experience with online shopping. In total, 76.7% of the participants in the current study reported that they engage in at least one online purchase per month, and 95.1% of the participants reported engaging in online purchasing at least once every 3 months.

### Theoretical Contributions and Practical Implications

A major deficiency of previous studies concerning monetary and social rewards is that the role of rewards as primers remains relatively unexamined. The current study used behavioral and ERP measures to examine reward processing across monetary and social reward types as primers, which had large differences from previous studies investigating social and monetary rewards (Izuma et al., 2008; Spreckelmeyer et al., 2009; Flores et al., 2015; Ait Oumeziane et al., 2017). Recent studies adapted MID and SID tasks in ERP studies to enable a comparison of social and reward processing in several stages (e.g., reward cue phrase, outcome anticipation phrase, and outcome evaluation/delivery phase) (Flores et al., 2015; Ait Oumeziane et al., 2017). The rewards in the MID or SID paradigms were given as feedback after the subjects made a choice. However, in the current study, the monetary or social rewards were offered before the participants made decisions. Thus, the participants could always gain the rewards regardless of their decisions. Therefore, the current study is the first to use reward for priming to study the difference between monetary and social rewards. Moreover, the paradigm of the rewards as primers was more consistent with reality as this paradigm represents an unethical manipulation offering consumers monetary rewards after they give a good rating on e-commerce platforms^1^ in China.

In addition, the aim of this paper was to open the black box of consumers’ brain in the decision-making process and introduce the ERP method to thoroughly explore the neurocognitive processes underlying monetary and social rewards as primers in making rating decisions in e-retailing. We found that the dynamic electrophysiological time course of consumers’ giving rating process could be divided into three main stages, with P2 reflecting early emotional evaluation, N2 reflecting conflict detection, and P3 reflecting the incentive effect. The ERP findings of P2, N2, and P3 could indicate that monetary rewards (such as discount coupons or cash coupons) as primers could compensate for the negative emotion caused by unsatisfactory products and had a greater motivation effect, while social rewards as primers may have no compensation effect for unsatisfactory products with a low incentive effect on consumers’ rating behavior in e-commerce.

The findings of the current study could be of great interest to online sellers. With the popularization of e-commerce, it is very possible to receive unsatisfactory goods due to the risk of uncertainty in online shopping. On the one hand, consumers grade products or service after their purchase, while on the other hand, they refer to online reviews before purchasing. Thus, it is necessary and important to understand the potential neural processes of how different reward types influence consumer cognition and emotion in making rating decisions, especially when consumers receive unsatisfactory products. A previous study indicated that enterprises should focus more on incorporating reward elements, such as monetary rewards with points and virtual goods and social rewards with badges and status, into mobile electronic commerce (Li, 2018). The results of this research illustrate the importance of giving cash/discount coupons to consumers in rating decision making. Online retailers should take advantage of monetary rewards to exert a positive influence on consumer rating behavior. However, notably, some marketing strategies involving monetary rewards are illegal, such as the strategy of returning cash/discount coupons if a consumer gives a five-star rating. Although previous research found that a strategy involving a monetary reward with related goals (illegal strategy) had a stronger effect on consumer rating behavior than monetary reward with no additional requirements (Wang et al., 2018), we suspect that there is a great negative effect on online sellers when the illegal marketing strategy is disclosed. Thus, e-retailers should be cautious when using monetary rewards in online marketing because it is conditional. Moreover, even if a marketing strategy involving a monetary reward as a primer is not illegal, its accompanying ethical issue should receive more attention. Monetary rewards could compensate more for consumer dissatisfaction derived from poor-quality products, further influencing consumer rating behavior. Therefore, monetary rewards make consumers disregard the real facts and induce an increase in online fake comments, which has a negative effect on the overall network environment.

In addition, the present results show that online reviews could be influenced by rewards, especially monetary rewards. Thus, online reviews can be manipulated to some extent, inducing an increase in fake comments. Consumers should be aware that it is very risky to depend only on comments when making decisions in online shopping. It is necessary for consumers to comprehensively consider various types of information, such as product sales, online reviews, and product descriptions, in purchase decisions.

### Limitations and Future Research

Several limitations in the current study need to be considered. First, considering that brain activities are sensitive and intricate, it is necessary for ERP experiments to follow strict environmental and equipment requirements. Therefore, the online purchase scenario in the current experiment was highly abstract. Although the discount coupons or cash coupons used as monetary reward stimuli were based on real-world scenarios, there were some differences from the actual online purchasing scenario. Second, the monetary rewards were reflected by cash/discount coupons, while the social rewards were reflected by greeting cards. To generalize the present findings, a wider range of monetary and social rewards with different intensities (e.g., badges or points on membership account) could be used as stimuli in future research. Third, the current ERP study compared monetary and social rewards at a brain level without considering the no-reward condition in the experimental design. Future research is needed to improve the paradigm and replicate the ERP findings with other behaviors, such as purchasing behavior or recommending behavior, and a greater sample size may increase the robustness of the current results. Moreover, most recruited participants were college students. Participants with more diverse backgrounds should be recruited to form a more comprehensive view of general brain activities during consumers’ rating decisions with rewards as primers.

## Conclusion

This research aimed to use behavioral and ERP measures to explore the priming effects of monetary and social rewards on rating decision making when consumers receive satisfactory or unsatisfactory products in e-retail. Using cash/discount coupons as a monetary reward and greeting cards as a social reward, the behavioral results showed that monetary rewards as primers for unsatisfactory products induced a more positive attitude and a longer reaction time than social rewards as primers, indicating that monetary rewards made it more difficult for the subjects to rate the unsatisfactory products than social rewards. The ERP results indicated that monetary rewards could compensate for unsatisfactory products during the early processing stage. The unsatisfactory products caused negative emotions that could be compensated by monetary rewards (but not social rewards) as reflected by the P2 component. Then, unsatisfactory products with monetary rewards were found to induce more decision conflict than those with social rewards as reflected by the N2 component. In the later controlled process, regardless of whether the products were satisfactory or unsatisfactory, monetary rewards caused more attention reallocation and were more motivating as reflected by the P3 component. To the best of our knowledge, the current study is among the first to use a reward as a primer to explore the differences between monetary and social reward types. Studying the different effects of monetary and social reward types on unsatisfactory products could help e-retailers understand the role of the two reward types in e-retail, especially when it is ineluctable that products or services are not completely satisfactory to consumers.

## Data Availability Statement

The datasets generated for this study are available on request to the corresponding author.

## Ethics Statement

The studies involving human participants were reviewed and approved by Internal Review Board of the Center for Management Decision and Neuroscience at Ningbo University. The patients/participants provided their written informed consent to participate in this study.

## Author Contributions

CW, WF, and JJ conceived and designed the experiments. CW, QS, and JJ performed the experiments. CW, WF, and XL analyzed the data. CW, QS, and XZ wrote and refined the article. All authors contributed to the article and approved the submitted version.

## Conflict of Interest

The authors declare that the research was conducted in the absence of any commercial or financial relationships that could be construed as a potential conflict of interest.

## Footnotes

1 Taobao.com
